# Pheochromocytoma and Paraganglioma in Neurofibromatosis type 1: frequent surgeries and cardiovascular crises indicate the need for screening

**DOI:** 10.1186/s40842-018-0065-4

**Published:** 2018-06-22

**Authors:** Elisabeth Joye Petr, Tobias Else

**Affiliations:** 1 Department of Internal Medicine, Division of Metabolism, Endocrinology and Diabetes, University of Michigan Health System, 24 Frank Lloyd Drive, Lobby C, Ann Arbor, MI 48105 USA; 2 Department of Internal Medicine, Division of Metabolism, Endocrinology and Diabetes, University of Michigan Health System, Medical Science Research Building II, Office # 2560e, 1150 West Medical Center Drive, Ann Arbor, MI 48109 USA

**Keywords:** Cardiovascular crisis, Hereditary tumor syndrome, Neurofibromatosis, Type 1, Paraganglioma, Pheochromocytoma, Screening

## Abstract

**Background:**

Pheochromocytomas and Paragangliomas (PCC/PGL) are rare endocrine tumors that are mostly benign, but often hormone producing, causing significant morbidity and mortality due to excess catecholamine secretion and cardiovascular crises. It is estimated that 30% of PCC/PGL are due to germline mutations, including Neurofibromatosis type 1 (*NF1*). There is little published data describing the phenotype of NF1-associated PCC/PGL and there are no established recommendations for PCC/PGL screening in NF1.

**Methods:**

We conducted a retrospective chart review of 17 patients with NF1-associated PCC/PGL who received care at a large academic referral center between the years of 1992–2016.

**Results:**

Average age of diagnosis was 42 years old. Both genders were equally affected. Average tumor size was 3.9 cm. Nine patients were hypertensive; one had orthostatic hypotension; three had tachycardia; the remaining two patients had normal BP and HR. Most tumors were benign, unilateral adrenal tumors that were hormonally active. Two had metastatic disease. Six patients experienced cardiovascular crises; three of which occurred during elective surgeries for neurofibroma removal, and a fourth occurred during labor and delivery.

**Conclusion:**

These data highlight the importance of screening for PCC/PGL in NF1, especially prior to surgical procedures and pregnancy, labor and delivery as these events can trigger a cardiovascular crisis. Screening is easily accomplished with plasma or urine free fractionated metanephrine levels.

**Electronic supplementary material:**

The online version of this article (10.1186/s40842-018-0065-4) contains supplementary material, which is available to authorized users.

## Background

Pheochromocytomas (PCC) and Paragangliomas (PGL) are rare endocrine tumors, occurring with an incidence of 0.8 per 100,000 [1]. Most of these tumors are benign, rather than malignant, however they can cause significant morbidity and mortality via catecholamine excess and resultant cardiovascular crises. A significant percentage (~ 30%) of those affected with PCC/PGL tumors harbor a germline mutation that predisposes them both to the development of PCC/PGL and also to other tumors unique to each particular inherited syndrome [1–5]. The most common known hereditary tumor syndromes that increase risk for PCC/PGL are Hereditary Paraganglioma Syndrome (*SDHx*), Neurofibromatosis Type 1 (*NF1*), von Hippel Lindau disease (*VHL*), Multiple Endocrine Neoplasia type 2 (MEN2, *RET*), *TMEM127*- and *MAX*-related hereditary pheochromocytoma, and Hereditary Leiomyomatosis and Renal Cell Cancer (HLRCC, *FH*) [6–8]. For MEN2 and VHL, there are recommendations to screen for PCC/PGL in mutation carriers (ie. annual metanephrine levels in MEN2 and annual metanephrine levels and review of adrenal glands on abdominal imaging in VHL) [9, 10]. For all other hereditary syndromes, there are no established screening recommendations for PCC/PGL. For rare conditions like PCC/PGL, often there is minimal evidence to guide the development of screening protocols. The value in screening at-risk gene mutation carriers for PCC/PGL is the opportunity to decrease morbidity and mortality through early disease detection and treatment.

Although in general PCC/PGL are rare tumors, NF1 is a common condition (prevalence of 1:3000 live births), and an estimated 5–7% of patients with NF1 will develop PCC/PGL in their lifetime [2]. NF1 has a high rate of de novo mutations (~ 50%), is inherited in autosomal dominant fashion, and has a variable clinical presentation [11]. The diagnosis of NF1 is made clinically in those who meet at least two of the following criteria: 6 or more café-au-lait macules, axillary or inguinal freckling, 2 or more neurofibromas or 1 plexiform neurofibroma, 2 or more Lisch nodules, an optic glioma, sphenoid or tibial bone dysplasia, or a first-degree relative with NF1 [12]***.***

Despite the fact that NF1 is common, and it is well established that NF1 increases the associated risk for PCC/PGL, there is little published data describing the phenotype of NF1-associated PCC/PGL, and there are no guidelines for the screening of PCC/PGL in NF1. In order to better characterize the phenotype of NF1-associated PCC/PGL, we conducted a retrospective study of patients with NF1 and PCC/PGL tumors who received care at a large academic referral center to describe their clinical presentation and disease course.

## Methods

We used electronic search algorithms based on diagnosis codes to identify patients with NF1 and PCC/PGL seen at the University of Michigan between 1992 and 2016. Charts of patients were reviewed to verify patients had confirmed diagnoses and then further reviewed for data gathering. We identified 17 patients with NF1 and PCC/PGL (Additional file 1: Table S1); 14 were referred with a diagnosis of PCC/PGL and three were diagnosed with PCC/PGL at our institution. This study was approved by the University of Michigan IRB (HUM00091004).

## Results

The average age at PCC/PGL diagnosis was 41 years with a range of 20–62 years old (Table 1). All had previously been diagnosed with NF1 by clinical criteria. Two had undergone genetic testing and had confirmed changes in the *NF1* gene. None of the patients were evaluated for other genetic syndromes predisposing to PCC/PGL. Both genders were affected equally, with nine male and eight female patients.

**Table 1:** Patient characteristics

Total number of patients	17
Gender (male/female)	9/8
Average age at diagnosis (years, range)	41 (20–62)
Means of diagnosis (biochemical/imaging/unknown)	5/11/1
Incidental Imaging finding (yes/no)	8/9
Presence of cardiovascular crisis (# patients)	6
Hypertension (yes/no/unknown)	9/8/0
Tachycardia, HR > 100 (yes/no/unknown)	4/5/5
Both HTN & Tachycardia (yes/no/unknown)	2/10/5
Number antihypertensive medications (average, range)	1 (0–4)
Elevated plasma or urine catecholamines or metanephrines (yes/no/unknown)	16/0/1
Level of metanephrine elevation (times upper limit of normal). (mean/median/range)	
UM UNM PM PNM	12.6/ 5.2/ (0.5–19.0) 4.6/ 2.7/ (0.6–3.5) 6.7/ 3.6/ (0.7–16.8) 3.2/ 2.0/ (1.4–10.2)
Elevations in both metanephrines & normetanephrines (yes/no/unknown)	10/1/6
MIBG scan (pos/neg/not done)	9/0/8

Abbreviations: *HTN* hypertension, *HR* heart rate, *UM* (urine metanephrine, reference range < 300 mcg/24 h), *UNM* (urine normetanephrine, reference range < 800 mcg/24 h), *PM* (plasma metanephrine, reference range < 0.5 nmol/L), *PNM* (plasma normetanephrine, reference range < 0.9 nmol/L), *MIBG* meta-iodobenzylguanidine

Nine PCC/PGL were identified during workup of hypertension or other suspicious symptoms. Eight were discovered incidentally on imaging performed for other reasons. Nine patients had hypertension, and one patient had low blood pressure with orthostatic hypotension. Five of the hypertensive patients were treated with antihypertensive medications; all were taking 1–2 medications except for one patient taking 4 antihypertensive medications. The remaining seven patients were normotensive, but three of these with normal blood pressures had tachycardia with HR elevated > 100, and two patients had borderline tachycardia with HR > 90.

Importantly, six patients experienced one or more cardiovascular crises including cardiac arrest, myocardial infarction (MI), intraoperative labile blood pressures, and extreme tachycardia during spinal anesthesia at the time of vaginal delivery. Three of these episodes occurred during elective surgeries for neurofibroma removal.

The average PCC/PGL tumor size was 3.9 cm, with a range of 1.5–6.8 cm (Table 2). There were 20 total tumors, 18 were located in the adrenal gland, one was an extra-adrenal abdominal PGL, and one was a head and neck PGL (HNPG). 13 patients had unilateral adrenal PCC. Three patients had bilateral PCC’s; two were synchronous and one was metachronous, occurring six years after diagnosis and treatment of the initial tumor. One of the patients with synchronous bilateral adrenal PCC also had a concurrent HNPG at the skull base.

**Table 2:** Tumor characteristics

Total number of tumors	20
Tumor location = Adrenal/ abdominal/ head & neck	18/1/1
Bilateral adrenal tumors (synchronous/metachronous)	3 (2/1)
Size (cm, range)	3.9 (1.5–6.8)
Malignant (yes/unknown)	2/1
Local recurrence	2

Two patients had local disease recurrence after initial tumor resection. One of these was the patient with extra-adrenal abdominal PGL, which recurred five years after initial resection and progressed to metastatic disease. The second patient had synchronous bilateral adrenal PCC that was managed with surgical removal of one total adrenal gland and partial removal of the contralateral adrenal gland, with local recurrence at the site of the partial adrenalectomy 11 years later.

Two patients had confirmed metastatic disease; a third patient had possible metastatic disease (nonspecific pulmonary nodules) at presentation, but did not follow up for completion of workup to determine etiology. The first had metastatic disease at the time of initial diagnosis, with the primary tumor a unilateral adrenal PCC. The second developed metastatic disease after recurrence of the extra-adrenal abdominal PGL. Both of these patients were in their fifth decade of life at the time of PCC/PGL diagnosis.

Biochemical data were available for 16 patients, all of whom had elevated plasma or urine metanephrine or catecholamine levels. Ten had elevations in metanephrine levels, with metanephrines more elevated above normal range than normetanephrines (Table 1). For the patients with adrenal PCC, plasma metanephrines were elevated 0.7–16.8 times upper limit of normal, plasma normetanephrines were elevated 1.4–10.2 times upper limit of normal, urine metanephrines were elevated 0.5–19.0 times upper limit of normal, and urine normetanephrines were elevated 0.6–3.5 times upper limit of normal. For the patient with the abdominal PGL, biochemical data on the original tumor was not available. At recurrence with metastatic disease 5 years later, plasma epinephrine level was elevated 16 times upper limit of normal. The patient with the HNPG had concomitant bilateral adrenal gland PCC, with urine metanephrines elevated 31 times upper limit normal and urine normetanephrines elevated 11 times upper limit of normal.

Nine patients underwent MIBG scans, all of which were positive (Table 1).

## Discussion

In summary, NF1-associated PCC/PGL presented at a similar age as reported for sporadic cases of PCC/PGL. The majority of NF1-associated PCC/PGL were benign unilateral adrenal tumors, which were biochemically active and positive on MIBG scans. Most patients had hypertension or tachycardia or both at the time of diagnosis. Although almost half of the PCC/PGL in this cohort (*n* = 8) were discovered incidentally on imaging, upon further investigation three of these patients had suggestive symptoms of PCC/PGL (tachycardia, palpitations, HTN urgency) and one had a preexisting adrenal nodule that had not been evaluated. Two of these patients had adrenal crises. This means that only the remaining four of the 17 subjects were asymptomatic at time of diagnosis The most notable finding in our study was the high incidence of cardiovascular crises in this cohort, affecting 6/17 patients; importantly, three of these crises occurred during neurofibroma removal surgeries, and one occurred during labor and delivery.

These data, particularly considering the high frequency of elective surgical procedures for patients with NF1, highlight the importance of PCC/PGL screening in the NF1 population, especially prior to situations that may trigger a crisis such as surgery or pregnancy, labor and delivery. This paradigm can be viewed similarly to the recommended management of patients with medullary thyroid carcinoma (MTC) who are screened for PCC/PGL prior to thyroidectomy surgery in order to reduce the risk for intraoperative cardiovascular crisis due to undiagnosed PCC/PGL in MEN2 syndrome [13].

There are some limitations to our findings. Our sample size was only 17 patients, however this is a relatively large cohort of patients compared to previously published reports of NF1-associated PCC/PGL. Our data were collected through retrospective chart review. Many of our patients received part of their care at other institutions, and we had limited details of prior care. Most of our patients did not undergo genetic testing for NF1 or other PCC/PGL associated genetic syndromes. Many patients with NF1 do not complete genotyping because NF1 is almost always diagnosed by clinical criteria. However, for the patients with extra-adrenal and head and neck PGL in this cohort, there might be a value in evaluation for other causes of PGL development, e.g. *SDHx* mutation.

At our institution, our practice is to evaluate patients with NF1 once a year including history, physical exam with blood pressure measurements (ie. Hypertension is often asymptomatic), and we offer plasma free fractionated metanephrine testing. It is especially important to screen for PCC/PGL in patients with NF1 who have hypertension or tachycardia. It is also important to emphasize to patients the importance of being screened for PCC/PGL prior to surgery or pregnancy, especially for those who do not participate in regular surveillance programs, as these situations can trigger a cardiovascular crisis. Patients with NF1 see a variety of specialists including primary care physicians, geneticists, neurologists, endocrinologists, obstetricians and gynecologists, surgeons, anesthesiologists, and both patient and physician awareness of all associated NF1 risks, including PCC/PGL, is vital for optimal multidisciplinary patient care.

Since almost all NF1-associated PCC/PGL appear to be biochemically active, screening with plasma or urine free fractionated metanephrine levels in the setting of a high pre-test probability should capture most cases. Most PCC/PGL were located in the adrenal gland, were amenable to complete surgical resection, and did not recur or progress to metastatic disease. The two patients with local disease recurrence had either incomplete resection of original adrenal PCC tumor, or an extra-adrenal abdominal PGL, which may behave in a more aggressive fashion. The two patients with metastatic disease were diagnosed at ages 44 and 48 years old suggesting that screening for PCC/PGL at earlier ages could decrease morbidity and mortality.

Recently, there have been two other retrospective studies regarding NF1-associated PCC/PGL with similar findings. A retrospective review of nine patients out of Montreal found frequent hypertension (6/9), with all tumors located in the adrenal gland, eight of which were unilateral and one patient with bilateral disease. There was no metastatic disease. All of the tumors were biochemically active. Four tumors were discovered during workup of suspicious symptoms; five were discovered incidentally on imaging. Two of the nine patients had hypertensive crises with pulmonary edema, one of which occurred during labor and delivery due to previously undiagnosed PCC/PGL [14].

A retrospective chart review of 41 patients from the Mayo Clinic between the years of 1959–2015 found hypertension in 39% of patients (16/41), a majority of patients with unilateral adrenal disease (32/41 or 78%), 17% (7/41) with bilateral adrenal tumors, and only 2 patients with extra-adrenal disease. 58% (21/41) were diagnosed with PCC/PGL after workup of suspicious symptoms; 11 were discovered incidentally on imaging. Two patients had labile blood pressures intraoperatively. Three had recurrent or metastatic disease: two of whom were young (Ages 14 and 21) and may have had more aggressive tumor type, and the third had an extra-adrenal PGL at age 58 [15].

## Conclusion

In summary, undiagnosed NF1-associated PCC/PGL poses a dangerous risk to patients, including cardiovascular crises such as labile intra-operative blood pressures, pregnancy and delivery complications, and severe complications including MI and cardiac arrest. Most NF1-associated PCC/PGL are detectable by biochemical evaluation. Therefore, we suggest consideration of screening adults with NF1 for PCC/PGL with plasma or urine free fractionated metanephrines every 1–2 years starting at age 18, especially in any NF1 patient with hypertension or tachycardia, and most importantly prior to any surgical procedures and pregnancy or delivery as these are times of increased risk for cardiovascular crisis. This approach has the potential to reduce the PCC/PGL-associated morbidity and mortality in the population of patients with NF1.

## Additional file

Additional file 1: **Table S1.** Summary of patient and tumor characteristics for NF1-associated PCC/PGL. (DOCX 24 kb)

## Abbreviations

BP: Blood pressure
FH: Fumarate Hydratase
HLRCC: Hereditary Leiomyomatosis and Renal Cell Cancer
HNPG: Head and Neck Paraganglioma
HR: Heart rate
HTN: Hypertension
MAX: Myc-associated factor X
MEN 2: Multiple Endocrine Neoplasia type 2
MI: Myocardial infarction
MIBG: meta-iodobenzylguanidine
NF1: Neurofibromatosis Type 1
PCC: Pheochromocytomas
PGL: Paragangliomas
RET: RET proto-oncogene
SDHx: Succinate Dehydrogenase
TMEM127: Trans-membrane encoding gene 127
VHL: von Hippel Lindau Syndrome
