# Supplementary material for: Pheochromocytoma and Paraganglioma in Neurofibromatosis type 1: frequent surgeries and cardiovascular crises indicate the need for screening
Source: Clin Diabetes Endocrinol. 2018 Jun 22;4:15. doi: 10.1186/s40842-018-0065-4 (PMC6013983; doi:10.1186/s40842-018-0065-4)
Supplement: Supplementary file 1 — Table S1. Summary of patient and tumor characteristics for NF1-associated PCC/PGL. (DOCX 24 kb) [file 40842_2018_65_MOESM1_ESM.docx]

**Table S1.** Summary of patient and tumor characteristics for NF1-associated PCC/PGL.

| **Patient** | **Gender** | **Age at PCC/PGL diagnosis (years)** | **PCC/PGL Presentation** | **Tumor Location** | **Tumor Size (cm)** | **BP** | **HR** | **Cardio-vascular Crisis** | **Biochemical**  **Testing (Times upper limit normal)** | **Scan** | **Recurrence, Metastatic Disease** |
| --- | --- | --- | --- | --- | --- | --- | --- | --- | --- | --- | --- |
| 1 | M | 62 | Incidental Imaging  (Abdominal MRI for unknown reason at outside hospital) | Right adrenal | 6 | 100/66 | x | Intra-operative labile blood pressure during PCC removal (not alpha blocked) | UM = 5.2  UNM = 3.5 | MRI, MIBG | no |
| 2 | F | 39 | Hypertension, Headaches, Tremor, Torso diaphoresis | Right adrenal | 6.8 | “hyper-tensive” | x | no | “Urine VMA elevated” | CT | no |
| 3 | M | 44 | Hypertension, palpitations, anxiety, night sweats | Extra-adrenal right abdominal paraganglioma over aortic bifurcation | 5 | “hyper-tensive” | x | no | x | CT, MIBG | 5 years after surgical resection, recurred locally in abdomen and progressed to metastatic disease in chest, abdomen, pelvis |
| 4 | M | 44 | Intra-operative labile blood pressure | Right adrenal | 4 | 110/60 | 95-105 | Intra-operative labile blood pressure | UM = 19.0  UNM = 1.8  PM = 16.8  PMN = 3.3 | CT, MIBG | no |
| 5 | F | 33 | Palpitations, diaphoresis, abdominal pain | Right adrenal | 4.3 | 137/79 | 109 | no | UM = 3.9  UNM = 1.7  PM = 3.5  PNM = 1.4 | MRI | no |
| 6 | F | 31 | Incidental Imaging  (CT for abdominal pain, N/V), however had had crisis during prior spinal anesthesia | Left adrenal | 4.2 | 120/65 | 105 | HR 200 during labor and delivery with spinal anesthesia | UM = 3.3  UNM = 0.8  PM = 3.7  PNM = 2.0 | CT, MIBG | no |
| 7 | F | 43 | Incidental Imaging  (CT for abdominal pain) | Left adrenal | 1.6 | 125/82 | 91 | no | PM = 1.1  PNM = 1.9 | CT | no |
| 8 | F | 20 | Hypertension, palpitations, headache, insomnia | Right adrenal | 1.5 | 163/89 | 92 | no | UM = 0.5  UNM = 0.6  PM = 0.7  PNM = 1.9 | CT | No |
| 9 | F | 27 | Intra-operative cardiac arrest | Left adrenal | 4.5 | “normal” | x | Intra-operative cardiac arrest | UM = 10  UMN = 4 | CT | no |
| 10 | M | 39 | Incidental Imaging  (CT for pulmonary nodules), however had been having palpitations | Left adrenal | 3.5 | 140/90 | 68 | no | “Urine epinephrine mildly elevated” | CT, MIBG | no |
| 11 | M | 34 | Incidental Imaging  (CT for abdominal pain), however had history of adrenal nodule | Right adrenal | 5.5 | 156/93 | 120 | no | PM = 12  PNM = 10 | CT, MIBG | no |
| 12 | M | 25 | Hypertension | Bilateral adrenal, synchronous | 3.3 (each side) | “hyper-tensive” | x | no | x | CT, MIBG | 11 years after left total adrenal-ectomy and right partial adrenal-ectomy, presented with Hyper-tensive stroke and local recurrence in right adrenal gland and with right periadrenal mass |
| 13 | M | 51 | Incidental Imaging  (MRI spine for back pain), however had uncontrolled HTN | Bilateral adrenal, metachronous, Left adrenal then right adrenal | 2.5 | 240/110 episodic | 88 | Hyper-tensive Urgency | PM = 2.1  PNM = 3.3 | MRI | 6 years after left adrenal-ectomy, right adrenal PCC discovered on CT abdomen screening protocol |
| 14 | F | 50 | Hypertension | Left adrenal | 3.2 | 150/92 | 52 | no | PM = 1.8  PNM = 1.9 | CT, MIBG | no |
| 15 | F | 51 | Recurrent Hypertensive emergencies (MI, cardiac arrest, labile BP in OR) | Bilateral adrenal (synchronous), and right skull base HNPG | Right 4.2, Left 3.1, HNPG 3.5 | 143/72, with recurrent hyper-tensive crises | 84 | Recurrent HTN emergency (MI, cardiac arrest, labile BP in OR) | UM = 31  UNM = 11 | MRI, MIBG | no |
| 16 | M | 57 | Incidental Imaging  (CT, MRI for unknown reason at outside hospital) | Left adrenal | 4.5 | 126/61 | 91 | no | "elevated urine catecholamines" | CT, MRI | no |
| 17 | M | 48 | Incidental Imaging  (CT abdomen concern for nephrolithiasis) | Left adrenal | 140 grams | 150/96 | 84 | no | "elevated urine metanephrines and normetanephrines" | CT | s/p left adrenal-ectomy, post-operative catecholamines remained elevated, 1 year later had abdominal pain, found to have metastatic disease in abdomen, liver, mediastinum |

Abbreviations: X = not documented or no info, HNPG = head and neck paraganglioma, OR = operating room, CT = computed tomography scan, MRI = magnetic resonance imaging scan, MIBG = meta-iodobenzylguanidine scan, BP = blood pressure, HR = heart rate, HTN = hypertension, Scan (Imaging scan), UM (urine metanephrine, reference range < 300 mcg/24hours), UNM (urine normetanephrine, reference range < 800 mcg/24 hours), PM (plasma metanephrine, reference range < 0.5 nmol/L), PNM (plasma normetanephrine, reference range < 0.9nmol/L), VMA = vanillylmandelic acid.
